# Alisertib promotes apoptosis and autophagy in melanoma through p38 MAPK-mediated aurora a signaling

**DOI:** 10.18632/oncotarget.22328

**Published:** 2017-11-06

**Authors:** Yuan-Yuan Shang, Ming Yao, Zhi-Wei Zhou, Jian-Cui, Li-Xia, Rong-Ying Hu, Ying-Yao Yu, Qiong-Gao, Biao-Yang, Yu-Xi Liu, Jie Dang, Shu-Feng Zhou, Nan-Yu

**Affiliations:** ^1^ Department of Dermatology, General Hospital of NingXia Medical University, Yinchuan, P.R.China; ^2^ Department of Pharmaceutical Sciences, College of Pharmacy, University of South Florida, Tampa, FL, USA; ^3^ Department of Burns and Plastic Surgery, General Hospital of NingXia Medical University, Yinchuan, P.R.China; ^4^ Department of Anesthesia, General Hospital of NingXia Medical University, Yinchuan, P.R.China; ^5^ Department of Medical Genetics and Cell Biology, Ningxia Medical University, Yinchuan, P.R.China

**Keywords:** alisertib, melanoma, AURKA, MAPK

## Abstract

We investigated the efficacy of Alisertib (ALS), a selective Aurora kinase A (AURKA) inhibitor, in melanoma. We found that ALS exerts anti-proliferative, pro-apoptotic, and pro-autophagic effects on A375 and skmel-5 melanoma cells by inhibiting p38 MAPK signaling. SB202190, a p38 MAPK-selective inhibitor, enhanced ALS-induced apoptosis and autophagy in both cell lines. ALS induced cell cycle arrest in melanoma cells through activation of the p53/p21/cyclin B1 pathway. Knockdown of p38 MAPK enhanced ALS-induced apoptosis and reduced ALS-induced autophagy. Inhibition of autophagy sensitized melanoma cells to ALS-induced apoptosis. These data indicate ALS is a potential therapeutic agent for melanoma.

## INTRODUCTION

Melanoma is a malignant cancer that arises from melanocytes. Approximately 132,000 individuals are diagnosed with melanoma each year. The 5-year survival rate of patients with metastatic melanoma is less than 20% [1]. Current treatments include surgery, chemotherapy, radiation therapy, immunotherapy, and targeted therapy. Targeted therapy is considered the most effective. The discovery of mutations in BRAF in melanoma lead to the development of vemurafenib, an orally available and well-tolerated selective inhibitor of BRAF V600E, for the treatment of patients with advanced disease [2]. However, there are few other biomarkers that can predict treatment response in melanoma patients and additional treatment strategies are needed [3].

Kinase inhibitors have demonstrated efficacy in various cancers including epithelial cell tumors with mutated forms of the epithelial growth factor receptor (EGFR) [4]. For example, an aurora kinase inhibitor has shown anticancer efficacy. There are three mammalian aurora kinases that belong to the aurora kinase family: aurora kinase A/B/C (AURKA/B/C) [5]. Aurora kinases play key roles in mitosis, and altered expression/activity can induce aneuploidy, genomic instability, and cell death [6]. At least 30 aurora kinase inhibitors are in various stages of preclinical and clinical development [7]. AURKA plays an important role in tumorigenesis because it normally regulates centrosome function, mitotic entry, chromosome segregation, and spindle assembly [8, 9]. Aberrant AURKA expression and activity have been demonstrated in several cancers including breast, bladder, colon, ovarian, liver, pancreatic, stomach, and esophageal cancer as well as melanoma [6, 10]. Increased AURKA expression was correlated with tumor differentiation and invasive capacity [11, 12].

**Figure 1:**
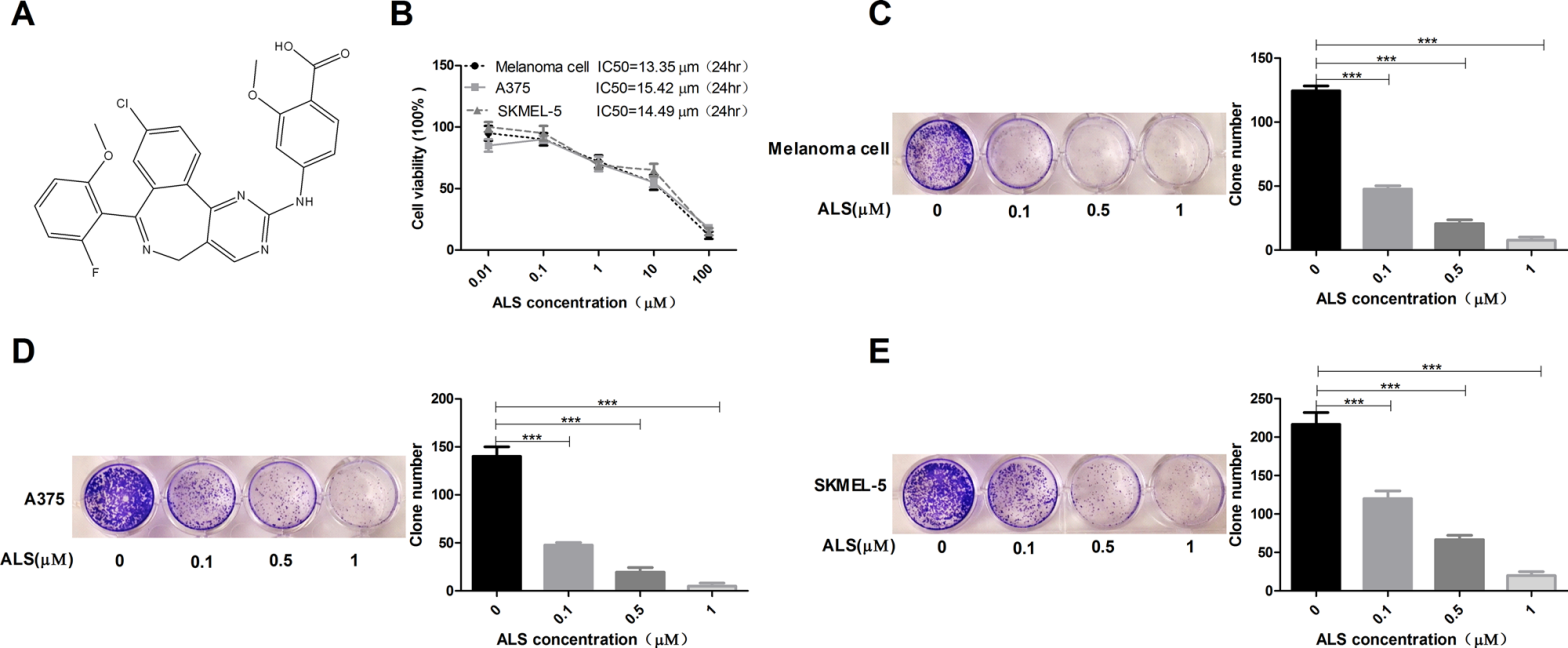
ALS decreases the viability and clonogenic capacity of melanoma cells **(A)** Chemical structure of ALS; **(B)** MTT assays of A375, skmel-5, and primary melanoma cell viability in response to treatment with ALS at concentrations ranging from 0.01 to 100 μM for 24 hours. IC_50_ values are shown; **(C-E)** Clonogenic capacity of A375, skmel-5, and primary melanoma cells as measured by colony formation assays following treatment with ALS at concentrations ranging from 0.01 to 100 μM for 24 hours. Data are expressed as the means ± SD. All experiments were repeated at least three times. (^*^p < 0.05, ^**^p < 0.01, ^***^p < 0.001).

Alisertib (ALS, MLN8237; Figure 1A) is an investigational small-molecule AURKA inhibitor. ALS has been investigated in Phase I and Phase II clinical trials and has demonstrated efficacy in patients with advanced-stage solid tumors and hematological malignancies [13]. However, ALS has not been investigated for the treatment of melanoma.

Mitogen-activated protein kinases (MAPKs), such as p38 MAPK, are activated in response to cellular and environmental stress (e.g. osmolarity, metabolism, DNA damage, heat shock, chemotherapeutic agents, ultraviolet irradiation, and oxidative stress). The p38 MAPKs regulate cell growth, cell cycle progression, apoptosis, and autophagy. However, the cellular responses are variable and dependent on the cell type, external stimulation, and experimental conditions [14]. Analyzing the role of p38 MAPK in the crosstalk between autophagy and apoptosis is challenging. We investigated the efficacy of ALS in melanoma. We characterized ALS-induced apoptosis and autophagy in melanoma A375 and skmel-5 melanoma cells.

## RESULTS

### ALS decreases the viability and clonogenic capacity of A375 and skmel-5 melanoma cells

We performed MTT assays to analyze the effects of ALS on A375 and skmel-5 cell viability. Treatment with 0.01 to 100 μM ALS for 24 hours inhibited the growth of A375, skmel-5, and primary human melanoma cells (Figure 1B). The IC_50_ values were 13.35, 15.42, and 14.49 μM for primary melanoma, A375, and skmel-5 cells, respectively. ALS also reduced the clonogenic capacity of the three cell types in a dose-dependent manner (Figure 1C-1E).

### ALS induces apoptosis and autophagy in A375 and skmel-5 cells through inhibition of the p38MAPK signaling pathway

**Figure 2:**
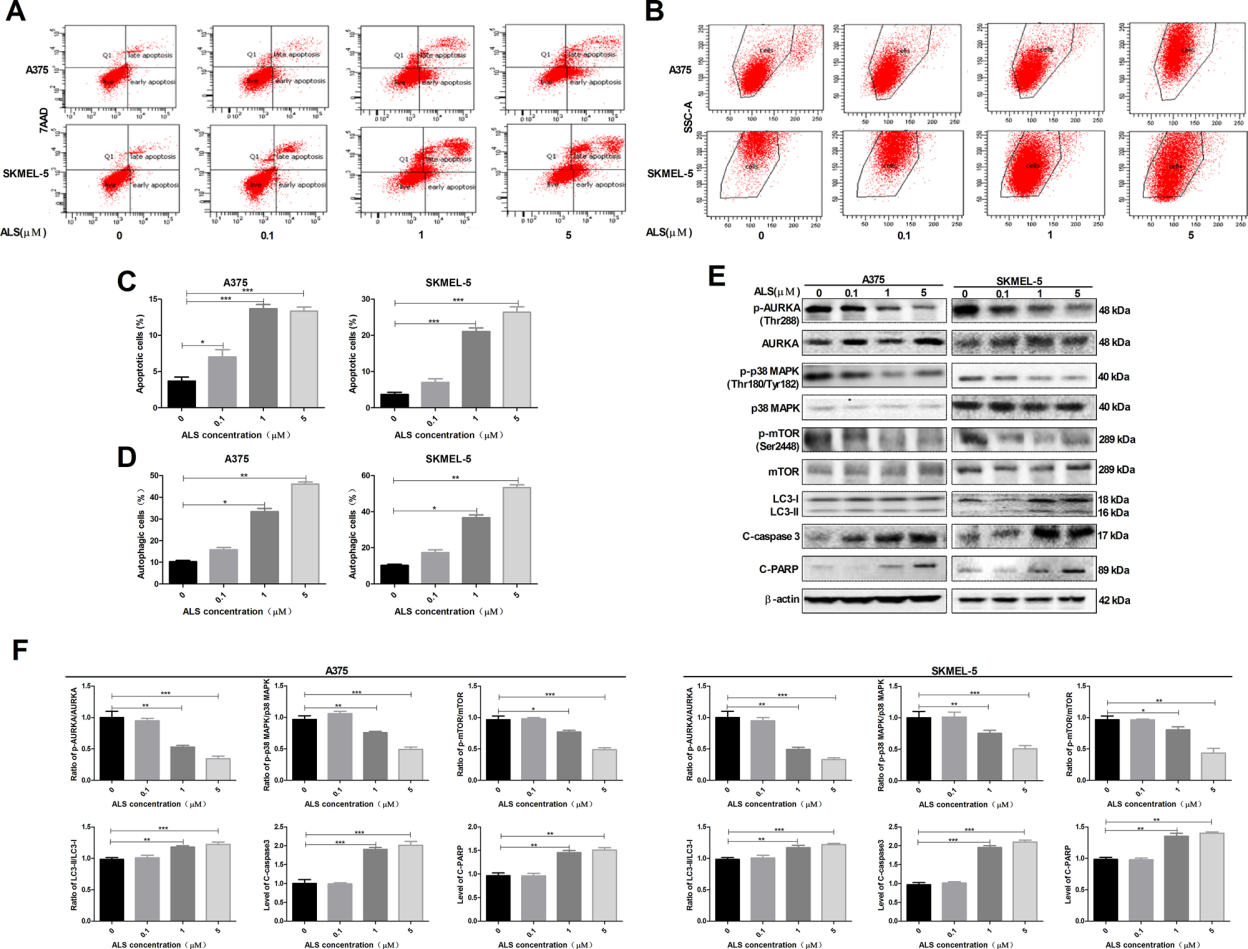
ALS induces apoptosis and autophagy in A375 and skmel-5 cells through inhibition of the p38 MAPK signaling pathway **(A)** Flow cytometry analysis of apoptosis in A375 and skmel-5 cells after treatment with ALS at concentrations ranging from 0 to 5 μM for 24 hours; **(B)** Flow cytometry analysis of autophagy in A375 and skmel-5 cells after treatment with ALS at concentrations ranging from 0 to 5 μM for 24 hours; **(C)** Quantification of apoptotic A375 and skmel-5 cells after treatment with AL; **(D)** Quantification of autophagic A375 and skmel-5 cells after treatment with ALS; **(E)** Western blot analysis of the levels of p38 MAPK signaling pathway components in A375 and skmel-5 cells after treatment with ALS at concentrations ranging from 0 to 5 μM; **(F)** Quantification of relative protein levels. Data are expressed as the means ± SD. All experiments were repeated at least three times. (^*^p < 0.05, ^**^p < 0.01, ^***^p < 0.001).

We next examined the effects of ALS on apoptosis and autophagy in A375 and skmel-5 cells using flow cytometry. Treatment of A375 cells with 0.1, 1, or 5 μM ALS for 24 hours resulted in an increase in the total percentage of apoptotic cells (early and late apoptosis) from 3.5% at baseline to 7.7%, 13.6%, and 13.2%, respectively. This corresponded to a 2.2-, 3.8-, and 3.7-fold increase, respectively, in the percentage of apoptotic cells relative to that of untreated control cells (*p* < 0.01 or *p* < 0.0001, Figure 2A and 2C). Treatment of skmel-5 cells with 0.1, 1, or 5 μM ALS for 24 hours resulted in an increase in the total proportion of apoptotic cells from 3.4% at baseline to 4.9%, 23.7%, and 27.2%, respectively. Treatment of skmel-5 cells with 1 and 5 μM ALS resulted in a 6.9- and 8-fold increase in the proportion of apoptotic cells, respectively, compared to untreated control cells (*p* < 0.0001, Figure 2A and 2C). Thus, ALS induced apoptosis in A375 and skmel-5 cells.

The percentage of autophagic cells at baseline was 9.7% and 10.1% for A375 and skmel-5 cells, respectively. Treatment of A375 cells with 1 or 5 μM ALS resulted in a 3.4- or 4.6-fold increase in the percentage of autophagic cells compared to untreated control cells (*p* < 0.001, Figure 2B and 2D). Similarly, treatment of skmel-5 cells with 1 or 5 μM ALS for 24 hours resulted in a 3.3- and 5.3-fold increase, respectively, in the percentage of autophagic cells (Figure 2B and 2D). These results indicated that ALS induced autophagy in both A375 and skmel-5 cells.

We next explored the mechanisms responsible for ALS-induced apoptosis and autophagy in A375 and skmel-5 cells. We examined phosphorylation of AURKA at Thr288 (p-AURKA) and p38 MAPK at Thr180/Tyr182 (p-p38 MAPK) following treatment with ALS. The levels of p-AURKA and p-p38 MAPK decreased in response to treatment with 0, 0.1, 1, and 5 μM ALS. However, ALS did not affect the total levels of AURKA or p38 MAPK (Figure 2E and 2F). We next evaluated the effects of ALS on m-TOR phosphorylation at Ser2448 (p-mTOR), a downstream effector of p38 MAPK, and LC3 expression in A375 and skmel-5 cells. LC3 is a marker of vesicle expansion and formation during autophagy [15]. LC3-I and p-mTOR levels decreased while LC3-II levels increased compared to baseline following treatment of A375 and skmel-5 cells with 1 or 5 μM ALS for 24 hours (Figure 2E and 2F). These results indicated that ALS induced LC3 activation and autophagy in A375 and skmel-5 cells. We also observed an increase in caspase-3 and PARP activation in A375 and skmel-5 cells following treatment with 1 or 5 μM ALS for 24 hours (Figure 2E and 2F). Thus, ALS induced apoptosis and autophagy in A375 and skmel-5 cells through inhibition of the p38MAPK signaling pathway.

### SB202190 enhances ALS-induced apoptosis and autophagy in A375 and skmel-5 cells

**Figure 3:**
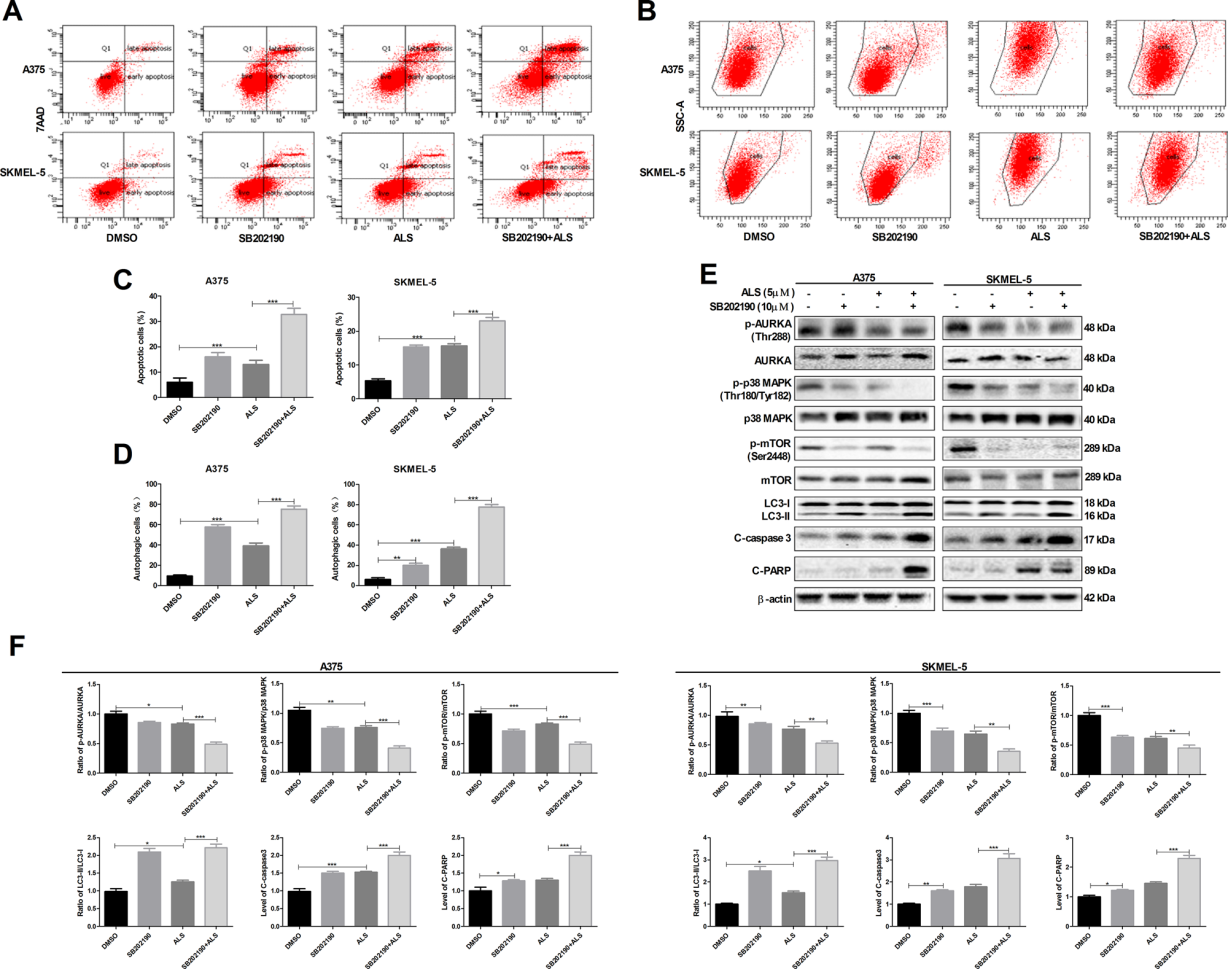
SB202190 enhances ALS-induced apoptosis and autophagy in A375 and skmel-5 cells **(A)** Flow cytometry analysis of apoptosis in A375 and skmel-5 cells after treatment with ALS, SB202190, or SB202190 + ALS for 24 hours; **(B)** Flow cytometry analysis of autophagy in A375 and skmel-5 cells after treatment with ALS, SB202190, or SB202190 + ALS for 24 hours; **(C)** Quantification of apoptotic A375 and skmel-5 cells after treatment with ALS, SB202190, or SB202190 + ALS; **(D)** Quantification of autophagic A375 and skmel-5 cells after treatment with ALS, SB202190, or SB202190 + ALS; **(E)** Western blot analysis of the levels of p38 MAPK signaling pathway components in A375 and skmel-5 cells after treatment with ALS, SB202190, or SB202190 + ALS; **(F)** Quantification of relative protein levels. Data are expressed as the means ± SD. All experiments were repeated at least three times. (^*^p < 0.05, ^**^p < 0.01, ^***^p < 0.001).

We next examined the effects of SB202190, a selective p38 MAPK inhibitor, on apoptosis and autophagy using flow cytometry. Treatment of A375 and skmel-5 cells with 10 μM SB202190 for 24 hours resulted in a 13.1% and 10.8% increase, respectively, in the total percentage of apoptotic cells (Figure 3A and 3C). Additionally, treatment of A375 and skmel-5 cells with 10 μM SB202190 and 5 μM ALS resulted in a 22.84% and 6.8% increase, respectively, in the total percentage of apoptotic cells compared to treatment with ALS alone (Figure 3A and 3C). Similarly, an 8.7% and 4.1% increase in the percentage of autophagic cells was observed in A375 and skmel-5 cells, respectively, compared to the baseline levels of 57.3% and 21.1%, respectively, following treatment with 10 μM SB202190 for 24 hours (Figure 3B and 3D). Treatment of A375 and skmel-5 cells with 10 μM SB202190 and 5 μM ALS resulted in a 36.8% and 44.4% increase in ALS-induced autophagy, respectively, compared to treatment with ALS alone (Figure 3B and 3D). These data demonstrated that SB202190 enhanced the levels of ALS-induced apoptosis and autophagy.

We also examined the levels of AURKA, p38 MAPK, mTOR, LC3, caspase-3, and PARP. SB202190 did not alter the levels of either p-AURKA or total AURKA compared to controls. Treatment of cells with ALS and SB202190 did not affect the levels of p-AURKA compared to cells treated with ALS alone (Figure 3E and 3F). Treatment of cells with SB202190 enhanced ALS-induced downregulation of p-p38 MAPK and p-mTOR (Figure 3E and 3F). Additionally, SB202190 enhanced the ALS-induced conversion of LC3-I to LC3-II resulting in an increase in the levels of cleaved caspase-3 and PARP in both A375 and skmel-5 cells (Figure 3E and 3F). These data indicated that inhibition of p38 MAPK enhanced ALS-induced apoptosis and autophagy in A375 and skmel-5 cells.

### ALS induces cell cycle arrest in A375 and skmel-5 cells

**Figure 4:**
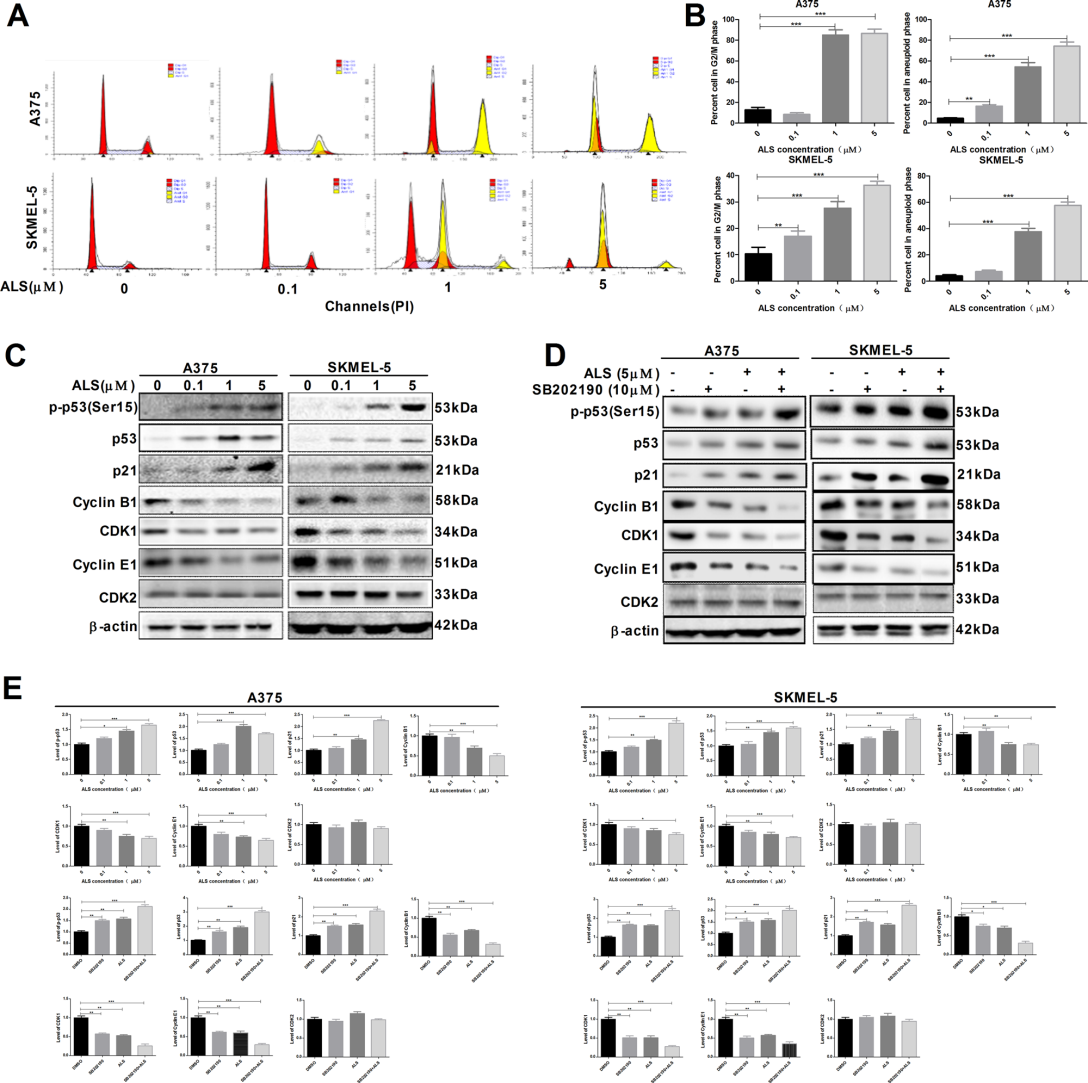
ALS induces cell cycle arrest in A375 and skmel-5 cells **(A)** Flow cytometry analysis of the cell cycle distribution of A375 and skmel-5 cells following treatment with ALS at concentrations ranging from 0 to 5 μM for 24 hours; **(B)** Quantification of A375 and skmel-5 cells in G2/M phase and aneuploid cells after treatment with ALS; **(C)** Western blot analysis of the levels of p53/p21/cyclin B1 pathway components in A375 and skmel-5 cells after treatment with ALS at concentrations ranging from 0 to 5 μM; **(D)** Western blot analysis of the levels of p53/p21/cyclin B1 pathway components in A375 and skmel-5 cells after treatment with ALS, SB202190, or SB202190 + ALS; **(E)** Quantification of the relative protein levels. Data are expressed as the means ± SD. All experiments were repeated at least three times. (^*^p < 0.05, ^**^p < 0.01, ^***^p < 0.001).

We examined changes in cell cycle phase after treatment of A375 and skmel-5 cells with 0.1, 1, or 5 μM ALS. ALS induced cell cycle arrest in the G2/M phase and increased the percentage of aneuploid cells in a dose-dependent manner in both cell lines (Figure 4A and 4B). Next, we explored possible mechanisms underlying ALS-induced cell cycle arrest. Treatment of A375 and skmel-5 cells with 0, 0.1, 1, and 5 μM ALS resulted in increased levels of p-p53 (Ser15), total p53, and total p21 in in a concentration-dependent manner (Figure 4C and 4E). We also evaluated the effects of ALS on cyclin B1, CDK1, cyclin E1, and CDK2 levels in A375 and skmel-5 cells. ALS treatment resulted in a decrease in cyclin B1, CDK1, and cyclin E1 levels in both cell lines compared to the levels in untreated control cells, but it did not alter CDK2 levels (Figure 4C and 4E). Additionally, the levels of p-p53, total p53, and total p21 increased, while the levels of cyclin B1, CDK1, and cyclin E1 decreased following ALS and SB202190 treatment compared to the levels in cells treated with ALS alone (Figure 4D and 4E). Thus, ALS induced G2/M cell cycle arrest in A375 and skmel-5 cells and increased the percentage of aneuploid cells through activation of the p53/p21/cyclin B1 pathway, and that SB202190 enhanced ALS-induced cell cycle arrest in melanoma cells.

### Knockdown of p38 MAPK enhances ALS-induced apoptosis and reduces ALS-induced autophagy in A375 and skmel-5 cells

**Figure 5:**
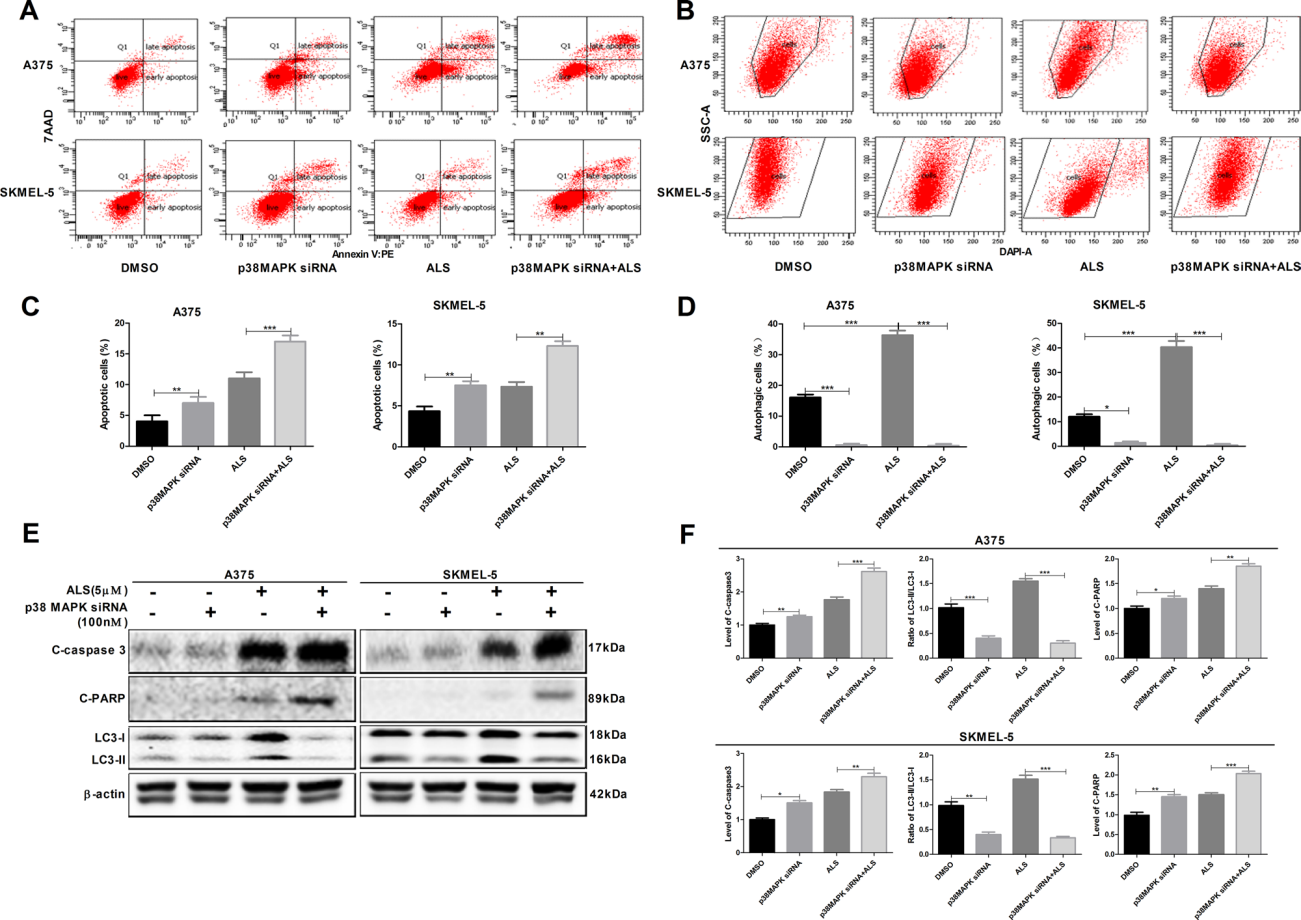
Signaling pathways involved in AURKA-stimulated p-p38 MAPK upregulation in A375 and skmel-5 cells **(A)** Flow cytometry analysis of apoptosis in A375 and skmel-5 cells after treatment with ALS, p38 MAPK siRNA, or p38 MAPK siRNA + ALS for 24 hours; **(B)** Flow cytometry analysis of autophagy in A375 and skmel-5 cells after treatment with ALS, p38 MAPK siRNA, or p38 MAPK siRNA + ALS for 24 hours; **(C)** Quantification of apoptotic A375 and skmel-5 cells after treatment with ALS, p38 MAPK siRNA, or p38 MAPK siRNA + ALS; **(D)** Quantification of autophagic A375 and skmel-5 cells after treatment with ALS, p38 MAPK siRNA, or p38 MAPK siRNA + ALS; **(E)** Western blot analysis of the levels of cleaved caspase-3, cleaved PARP, LC3-I, and LC3-II in A375 and skmel-5 cells after treatment with ALS, p38 MAPK siRNA, or p38 MAPK siRNA + ALS; **(F)** Quantification of the relative protein levels. Data are expressed as the means ± SD. All experiments were repeated at least three times. (^*^p < 0.05, ^**^p < 0.01, ^***^p < 0.001).

We examined the effects of p38 MAPK knockdown by siRNA on apoptosis and autophagy in A375 and skmel-5 cells using flow cytometry. Silencing p38 MAPK expression resulted in an increase in apoptosis and a decrease in autophagy in both cell lines (Figure 5). An increase in the percentage of apoptotic cells and a decrease in the percentage of autophagic cells was observed in response to p38 MAPK knockdown in A375 and skmel-5 cells compared to cells treated with ALS alone. We also found that silencing p38 MAPK resulted in an increase in the levels of cleaved caspase-3 and PARP compared to controls, and a decrease in the levels of LC3-II. Additionally, cells treated with the p38 MAPK-specific siRNA and ALS exhibited a reduction in LC3-II levels and an increase in cleaved caspase-3 and PARP levels compared to cells treated with ALS alone (Figure 5E and 5F). These data revealed that knockdown of p38 MAPK enhanced ALS-induced apoptosis and reduced ALS-induced autophagy in melanoma cells.

### Inhibition of autophagy enhances ALS-induced apoptosis in A375 and skmel-5 cells

**Figure 6:**
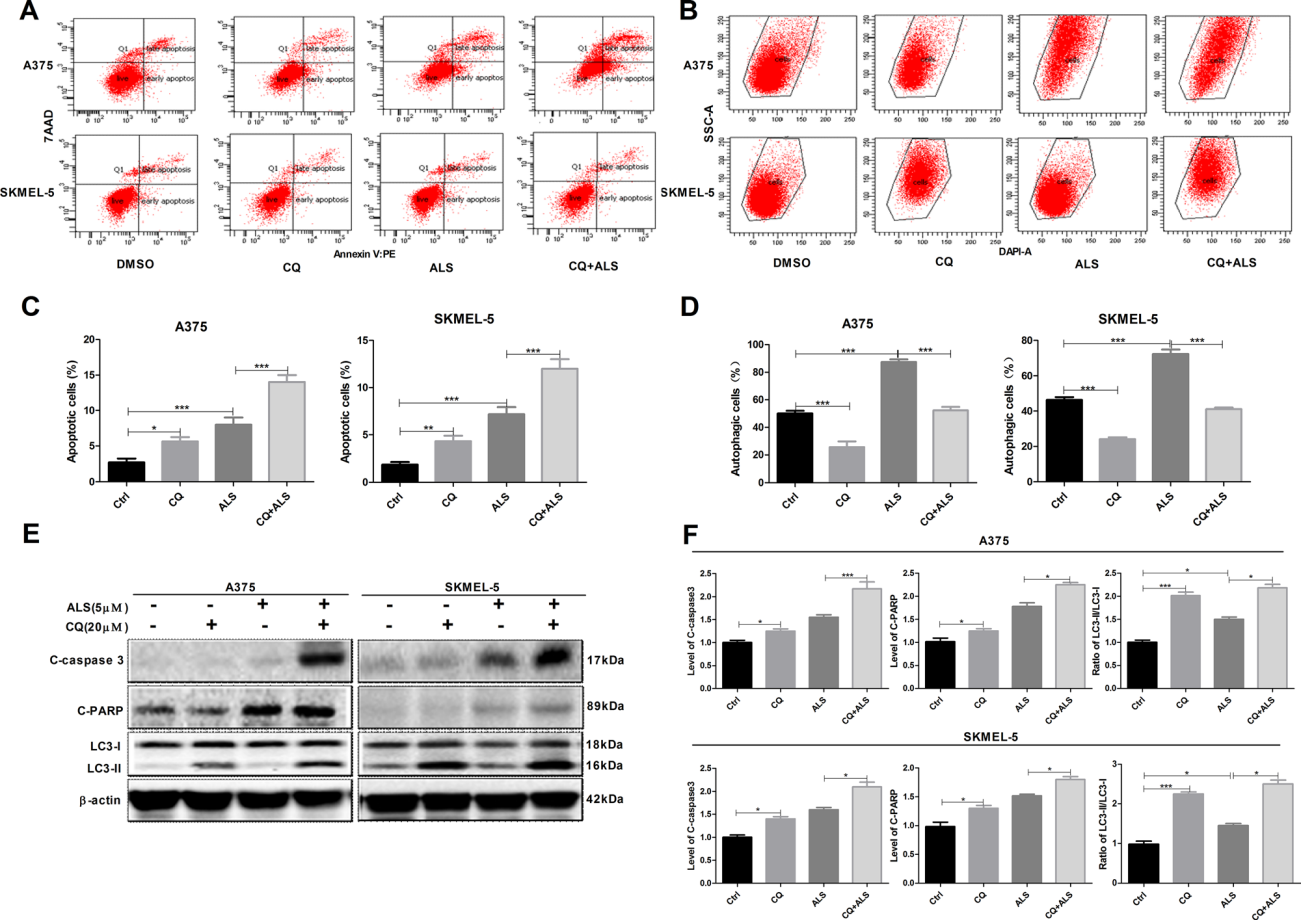
Inhibition of autophagy sensitizes A375 and skmel-5 cells to ALS-induced apoptosis **(A)** Flow cytometry analysis of apoptosis in A375 and skmel-5 cells after treatment with ALS, CQ, or CQ + ALS for 24 hours; **(B)** Flow cytometry analysis of autophagy in A375 and skmel-5 cells after treatment with ALS, CQ, or CQ + ALS for 24 hours; **(C)** Quantification of apoptotic A375 and skmel-5 cells under treatment with ALS, CQ or CQ + ALS. **(D)** Quantifications of autophagic A375 and skmel-5 cells after treatment with ALS, CQ, or CQ + ALS; **(E)** Western blot analysis of cleaved caspase-3, cleaved PARP, LC3-I, and LC3-II levels in A375 and skmel-5 cells after treatment with ALS, CQ, or CQ + ALS; **(F)** Quantification of the relative protein levels. Data are expressed as the means ± SD. All experiments were repeated at least three times. (^*^p < 0.05, ^**^p < 0.01, ^***^p < 0.001).

To investigate the role of autophagy in the ALS-induced apoptosis, A375 and skmel-5 cells were treated with CQ and autophagy analyzed by flow cytometry. A375 and skmel-5 cells treated with 20 μM CQ alone for 24 hours exhibited a decrease in the total percentage of autophagic cells compared to untreated control cells. However, an increase in the percentage of apoptotic cells was observed (Figure 6A-6D). In comparison to cells treated with ALS alone, the total percentage of apoptotic cells increased, which the percentage of autophagic cells decreased following treatment with ALS and CQ. Treatment of A375 and skmel-5 cells with CQ resulted in a decrease in LC3-II levels but an increase in cleaved caspase-3 and PARP levels (Figure 6E and 6F). LC3-II levels decreased, while caspase-3 and PARP levels increased in cells treated with ALS and CQ compared to cells treated with ALS alone.

### AURKA promotes activation of the p-p38 MAPK signaling pathway in A375 and skmel-5 cells

**Figure 7:**
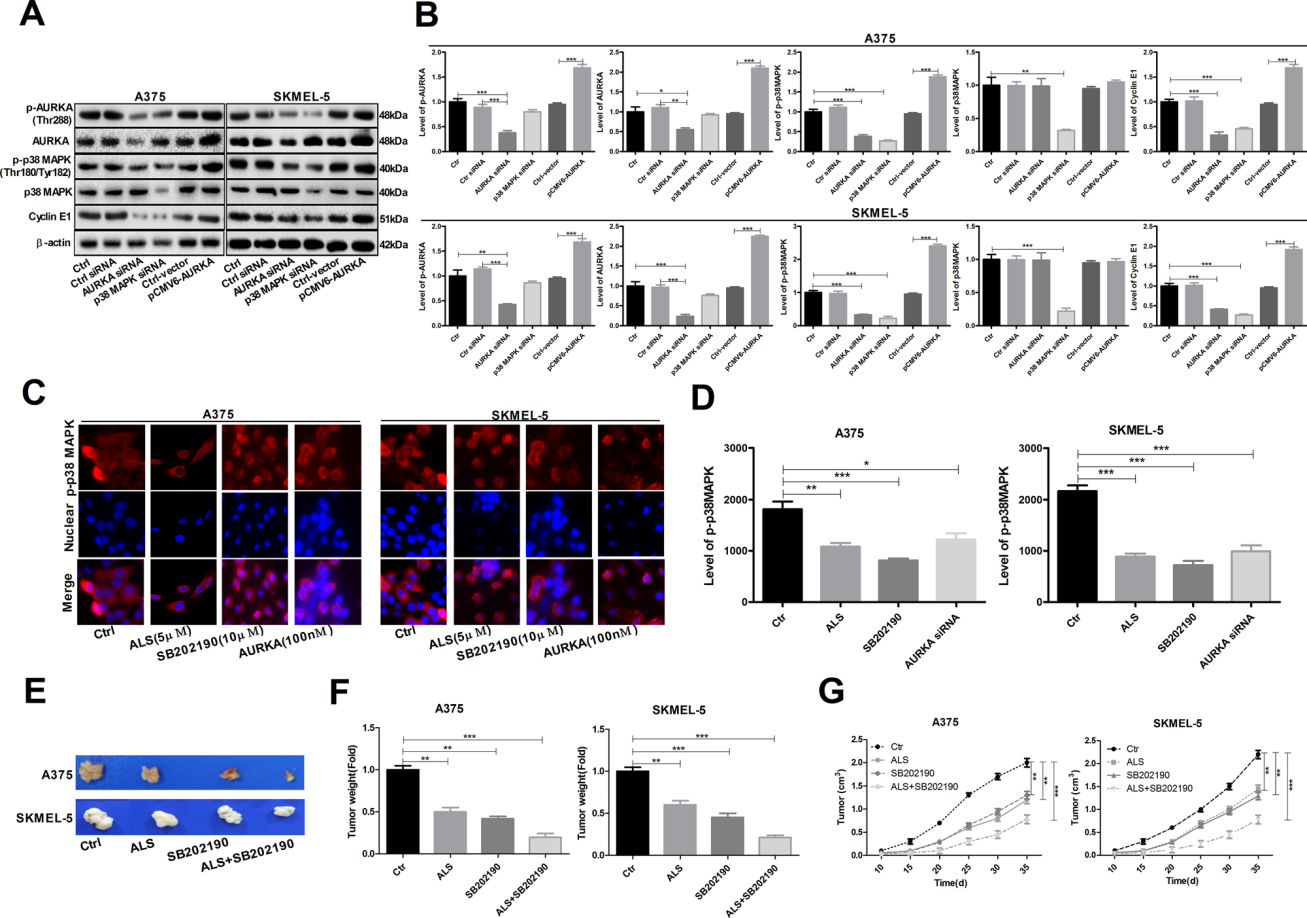
AURKA-stimulated upregulation of p-p38 MAPK levels in A375 and skmel-5 cells **(A)** Western blot analysis of p38 MAPK signaling pathway components in A375 and skmel-5 cells; **(B)** Quantification of the relative protein levels; **(C)** Immunofluorescence analysis of p-p38 MAPK levels in A375 and skmel-5 cells; **(D)** Quantification of the relative immunofluorescence intensities; **(E)** Images of tumor cell morphology in the different treatment groups; **(F)** Tumor weights in the different treatment groups; **(G)** Tumor growth curves. Data are expressed as the means ± SD. All experiments were repeated at least three times. (^*^p < 0.05, ^**^p < 0.01, ^***^p < 0.001).

Reduced levels of p-AURKA, AURKA, p-p38 MAPK, and cyclin E1 were observed following transfection of A375 and skmel-5 cells with AURKA- or p38 MAPK-specific siRNAs compared to cells transfected with control siRNAs (Figure 7A and 7B). We also examined the levels of p-p38 MAPK in A375 and skmel-5 cells by immunofluorescence. Treatment of A375 and skmel-5 cells with 5 μM ALS, 10 μM SB202190, or 100 nM AURKA siRNA resulted in a decrease in p-p38 MAPK levels (Figure 7C-7E).

### ALS inhibits tumorigenesis by suppressing p38 MAPK signaling

We investigated the role of AURKA in tumorigenesis in a mouse model of melanoma. Both ALS (5 μM) and SB202190 (10 μM) inhibited tumorigenesis. The greatest effects were observed in the ALS + SB202190 group compared to the control group (Figure 7E-7G), suggesting that ALS inhibits tumorigenesis by suppressing p38 MAPK signaling.

## DISCUSSION

AURKA has emerged as a therapeutic target in various solid and hematologic cancers. In this study, we investigated the effects of ALS, a potent and selective inhibitor of AURKA, on melanoma cells. MAPKs have important roles in cancer development and progression [16]. There are six distinct MAPK groups in mammals: JNK1/2/3, ERK1/2, ERK3/4, ERK5, ERK7/8, and the p38 isoforms a/b/c (ERK6)/d [17]. A variety of stimuli induce activation of MAPK pathways including genotoxic agents, ultraviolet irradiation, G-protein-coupled receptors, oxidative stress, inflammation, growth factors, and cytokines. Activation of MAPK signaling results in various cellular responses including senescence, proliferation, and differentiation [18, 19]. Apoptosis and autophagy play important roles in normal cell physiology and homeostasis. Although apoptosis is the principal mechanism of programmed cell death in mammalian cells, autophagy also can have a pro-death or a pro-survival role depending on the strength of specific stimuli and on cell type [20, 21]. Although there is crosstalk between the pathways leading to apoptosis and autophagy, the stimuli and underlying mechanisms have not been elucidated. MAPKs such as p38 MAPK likely play a key role in the crosstalk between apoptosis and autophagy. We explored the role of AURKA-stimulated p38 MAPK signaling in regulating the balance of apoptosis and autophagy in A375 and skmel-5 cells.

p38 MAPK plays key roles in cell cycle arrest, apoptosis, differentiation, and growth inhibition [22]. It is proposed to act as a tumor suppressor. Many chemotherapeutic agents require p38 MAPK activity for the induction of apoptosis [23]. For example, cyclophosphamide and anthocyanins promote apoptosis by activating p38 MAPK in breast and colon cancer cells [24, 25]. We found that ALS promoted apoptosis and inhibited autophagy in A375 and skmel-5 melanoma cells through inhibition of p38 MAPK activation.

Cyclooxygenase-2 (COX-2) overexpression was shown to trigger resistance to apoptosis in both HT-29 and DU145 cells through activation of p38 MAPK [26]. Another study demonstrated that cetaxel-induced resistance to apoptosis was enhanced by p38 MAPK phosphorylation [27]. Thus, p38 MAPK may have a dual role in the regulation of autophagy, both as a positive and negative regulator. Oridonin was reported to induce autophagy through activation of p38 MAPK [28]. Additionally, p38 MAPK contributed to ZnPPIX-induced autophagy, which was dependent upon Beclin 1 [29], and also negatively regulated autophagy. TNF-α promotes necroptotic and autophagic cell death through inhibition of p38 MAPK [30], indicating that under certain conditions, p38 MAPK can mediate resistance to apoptosis and autophagy. We demonstrated that ALS induced apoptotic cell death by decreasing p38 MAPK phosphorylation in melanoma cells.

Treatment of A375 and skmel-5 cells with ALS also resulted in cell cycle arrest in the G2/M phase and a concentration-dependent increase in the percentage of aneuploid cells (Figure 4). Treatment of the cells with SB202190 resulted in an increase in the levels of p-p53, total p53, and p21, and a decrease in the levels of cyclin B1, CDK1, and cyclin E1. These data indicated that ALS induced cell cycle arrest and an increased the percentage of aneuploid cells through activation of the p53/p21/cyclin B1 pathway.

Silencing p38 MAPK in A375 and skmel-5 cell lines resulted in an increase in apoptosis and a decrease in p-p38 MAPK levels. However, the levels of total AURKA and p-AURKA were unaffected. These data indicate AURKA has a critical role in regulating p-p38 MAPK levels as well as apoptosis and autophagy in melanoma cells. Interestingly, silencing p38 MAPK induced apoptosis but suppressed ALS-induced autophagy in A375 and skmel-5 cells. Therefore, p38 MAPK may regulate autophagy both positively and negatively in melanoma cells. A reduction in total p38 MAPK levels could lead to genomic instability and delayed activation or inhibition of autophagy in response to stress.

The four different p38 MAPKs (α, β, γ, and δ) exhibit distinct expression patterns and affinities for upstream activators and downstream effectors [31]. SB202190 is widely used to assess the physiological roles of p38α and p38β MAPKs. Reduced p38α MAPK activity led to an increase in autophagy in colorectal cancer cells through upregulation of the human homolog of Atg8. SB202190-mediated autophagy was dependent upon p38α MAPK blockade [32]. We found that ALS-induced autophagy was suppressed by knockdown of p38 MAPK, indicating that ALS induces apoptosis and autophagy via p38 MAPK signaling.

We investigated whether ALS-induced autophagy promote cell survival or cell death using CQ, which disrupts lysosomal function and inhibits autophagy by interfering with the acid-dependent degradation of proteins within autophagosomes [33]. CQ enhanced ALS-induced apoptosis and inhibited ALS-induced autophagy, suggesting that ALS-induced autophagy might attenuate apoptosis. Tiwari et al. reported that inhibition of autophagy resulted in a switch to apoptotic cell death [34]. Simone et al. found that chemotherapeutic- or siRNA-mediated inhibition of the p38 MAPK pathway resulted in cell cycle arrest and autophagic cell death in colorectal cancer cells [35]. Chiacchiera et al. demonstrated that inhibition of p38 MAPK induced autophagy. However, prolonged inhibition of p38 MAPK resulted in autophagic cell death [36]. Another study found that p38 MAPK played a vital role in the switch from autophagy to apoptosis. High p38 MAPK expression resulted in autophagy in MS-275-induced human colon cancer cells, while low expression resulted in a decrease in apoptosis [37]. Thus, p38 MAPK may mediate crosstalk between apoptotic and autophagic pathways, although the mechanisms by which p38 MAPK regulates the balance between apoptosis and autophagy in response to chemotherapeutic agents are not yet clear.

Our data suggest that ALS-induced autophagy has a protective effect on cell survival. However, inhibition of p38MAPK suppressed both basal and ALS-induced autophagy in melanoma cells. Thus, further studies are required to elucidate the role of p38 MAPK in chemotherapy-induced autophagy.

## MATERIALS AND METHODS

### Reagents

ALS (MLN8237) was purchased from Selleckchem Inc. (Houston, TX, USA). Dulbecco’s Modified Eagle’s Medium (DMEM), DMEM nutrient mixture F12 (DMEM/F12), Dulbecco’s phosphate-buffered saline (PBS), dimethyl sulfoxide (DMSO), heat-inactivated fetal bovine serum (FBS), RNase A, propidium iodide, and thiazolyl blue tetrazolium bromide (MTT) were purchased from Sigma-Aldrich Inc. (St Louis, MO, USA). Chloroquine (CQ, an autophagy inhibitor) and SB202190 (p38 MAPK inhibitor) were purchased from InvivoGen Inc. (San Diego, CA, USA). The PE Annexin V Apoptosis Detection Kit was obtained from BD Biosciences Inc. (San Jose, CA, USA). The Cyto-ID® autophagy detection kit was sourced from Enzo Life Sciences Inc. (Farmingdale, NY, USA). Polyvinylidene difluoride membranes were bought from EMD Millipore Inc. (Billerica, MA, USA). Primary antibodies against human cleaved caspase-3, PARP, microtubule-associated protein 1A/1B-light chain 3 (LC3-I), p38 MAPK, p38 MAPK phosphorylated at Thr180/Tyr182, mTOR, mTOR phosphorylated at Ser2448, AURKA, and AURKA phosphorylated at Thr288, as well as p38 MAPK siRNA I #6564 and AURKA/AIK siRNA I #8883 were all purchased from Cell Signaling Technology Inc. (Beverly, MA, USA), The pCMV6-AURKA construct was purchased from Origene (No. PC212018, Rockville, MD, USA).

### Cell lines and culture

Two melanoma cell lines, A375 and skmel-5, were obtained from the American Type Culture Collection (ATCC, Manassas, VA, USA) and cultured in DMEM (A375 cells) or DMEM/F12 (skmel-5 cells) media supplemented with 10% heat-inactivated FBS and 1% penicillin/streptomycin. Primary melanoma cells were isolated from patient tissue. The cells were maintained in a 5% CO_2_ humidified incubator at 37°C. ALS and SB202190 were dissolved in DMSO and CQ was dissolved in water as stock solutions. Stock solutions were then diluted in culture media for experiments. The final concentration of DMSO was 0.1% for all treatments including controls.

### Cell viability and colony formation assays

The effects of ALS on A375 and skmel-5 cell proliferation were examined using MTT and colony formation assays. For MTT assays, A375 and skmel-5 cells were seeded into 96-well plates at a density of 8,000 cells/well in 100 μL of media. After 24 hours, the cells were treated with ALS at concentrations ranging from 0.1 to 100 μM for 24 hours. Following ALS treatment, 10 μL of MTT stock solution (5 mg/mL) was added to each well and the cells incubated for 4 hours. The media was then removed and 100 μL of DMSO added to each well. Cell viability was measured by the reduction of MTT after 10 minute incubation at 37°C. The absorbance at 560 nm (MTT formazan) and 670 nm (background) was measured using a Synergy™ H4 Hybrid microplate reader (BioTek, Winooski, VT, USA). IC_50_ values were calculated using the relative viability over the ALS concentration curve. All experiments were performed in triplicate.

For colony formation assays, A375 and skmel-5 cells were seeded into 12-well plates at a density of 300 cells/well and incubated for 24 hours. Following the incubation, the cells were treated with ALS at concentrations of 0, 0.1, 0.5, or 1 μM for 24 hours. The media was removed and 2 mL of fresh culture media added to each well. The cells were incubated for 14 days. The media was replaced every 3 days. Once colonies were visible, the cells were washed twice with PBS and fixed with 5 mL of methanol for 15 minutes. The methanol was removed and the cells stained with Giemsa for 10 to 30 minutes. The staining solution was then removed and the cells rinsed with water. After air-drying, the cells were imaged. Colonies with more than 50 cells were counted under a microscope and the rate of colony formation was calculated.

### Quantification of apoptosis

Apoptosis was analyzed following treatment of A375 and skmel-5 cells with 0.1, 1, or 5 μM ALS for 24 hours using the PE Annexin V Apoptosis Detection Kit. The mechanisms underlying ALS-induced apoptosis were evaluated by pretreating the cells with 20 μM CQ or 10 μM SB202190 for 1 hour, and then incubating the cells with 5 μM ALS for an additional 24 hours. The cells were subsequently harvested, washed with cold PBS, and stained with a combination of 5 μL Annexin V: PE and 5 μL 7-amino-actinomycin D in a 100 μL solution in the dark for 15 minutes at room temperature. Finally, 150 μL of 1× binding buffer was added to each tube and apoptosis quantified using flow cytometry.

### Autophagy analysis

A375 and skmel-5 cells were treated with 0.1, 1, or 5 μM ALS for 24 hours and autophagy analyzed using flow cytometry. The mechanisms underlying ALS-induced autophagy were evaluated by pretreating the cells with 20 μM CQ or 10 μM SB202190 for 1 hour, and then treating them with 5 μM ALS for an additional 24 hours. The cells were subsequently trypsinized, washed with 1× assay buffer, and resuspended in 250 μL of solution containing 2.5 μL Cyto-ID Green stain for 30 minutes at 37°C in the dark. Following the incubation, the cells were washed with 1× assay buffer and resuspended in 250 μL of fresh 1× assay buffer containing 5% FBS. Finally, the cells were analyzed by flow cytometry using the fluorescein isothiocyanate channel. A total of 20,000 events were analyzed.

### Cell cycle analysis

A375 and skmel-5 cells were seeded in 60-mm tissue culture dishes at a density of 2.5 × 10^5^ cells/dish and cultured overnight. The following day, the cells were treated with ALS at concentrations of 0.01, 0.1, or 0.5 μM for 24 hours. The cells were then trypsinized and fixed in 3 mL of 70% ethanol at −20°C overnight. After fixation, the cells were harvested and resuspended in 1 mL of PBS containing 1 mg/mL of RNase A and 50 μM/mL of propidium iodide. The cells were incubated in the dark for 30 minutes at room temperature and cell cycle phase analyzed by flow cytometry.

### Western blotting analysis

A375 cells and skmel-5 cells were cultured with various concentrations of ALS for 24 hours, and whole cell lysates generated. The samples were subjected to 7–12% sodium dodecyl sulfate polyacrylamide gel electrophoresis on mini-gels. Proteins were electrophoretically transferred to polyvinylidene difluoride membranes at 400 mA for 2 hours at 4°C. Membranes were treated with 5% skim milk for 1 hour at room temperature to block non-specific binding. The membranes were incubated with the indicated primary antibodies overnight at 4°C followed by the appropriate secondary antibodies for 2 hours. Proteins were visualized using a Bio-Rad ChemiDoc™ XRS system (Bio-Rad Laboratories Inc., Hercules, CA, USA). Image Lab 3.0 (Bio-Rad Laboratories Inc.) was used for analysis. The protein levels were normalized to the matching densitometric values of β-actin.

### RNA interference

Cells were transfected with RNA oligonucleotides using Lipofectamine 2000 according to the manufacturer’s instructions. A375 and skmel-5 cells were transfected with the negative control siRNA, 100 nM p38 MAPK siRNA, or 100 nM AURKA siRNA in Opti-MEM. After a 4 hour incubation, the Opti-MEM™ was replaced with complete media and the cells incubated for 48–72 hours. Cell samples were then collected for further analysis.

### Immunofluorescence

A375 and SKMEL-5 were seeded in four-well chamber slides and pretreated with 5 μL of ALS, 10 μM SB202190, and 100 nM AURKA siRNA. Cells were fixed with 4% formaldehyde, rinsed with PBS, and then blocked in 5% bovine serum albumin (BSA) for 1 hour at room temperature. The primary antibody, rabbit anti-phosphorylated p38 MAPK (1:200), was added to the 5% BSA, and the samples incubated at 4°C overnight. The slides were washed three times with 1% PBS (5 minutes/wash) and then incubated with a goat anti-rabbit rhodamine-conjugated secondary antibody (1:200) for 2 hours at room temperature in the dark. The samples were mounted on cover slips with Gel/Mount and sealed with clear nail polish. The expression of phosphorylated p38 MAPK was analyzed using a TCS SP2 laser scanning confocal microscope (Leica, Wetzlar, Germany) and a standard fluorescein isothiocyanate filter (647 nm).

### *In vivo* tumorigenesis

All animal experiments were approved by Animal Ethics Committee of the General Hospital of NingXia Medical University and were performed in accordance with the protocol and requirements of the Animal Ethics Committee of NingXia Medical University. SPF grade male BALB/c nude mice were purchased from the Institute of Zoology, Chinese Academy of Sciences. A375 and skmel-5 cells were re-suspended in 0.1 mL PBS and subcutaneously injected into 4-week-old male nude mice (1 × 10^7^ cells/mouse). Mice were sacrificed 35 days after injection and the weights of all tumors measured.

### Statistical analysis

The data are presented as the means ± standard deviations. Comparisons between multiple groups were performed using one-way analyses of variance followed by Tukey’s multiple comparison tests. A *p* < 0.05 was considered statistically significant. Assays were performed at least three times.

## Abbreviations

(ALS): Alisertib
(AURKA): a selective aurora kinase A
(MAPKs): Mitogen-activated protein kinases
(ATCC): American Type Culture Collection
(COX-2): Cyclooxygenase-2
